# Identification of white campion (*Silene latifolia*) guaiacol *O*-methyltransferase involved in the biosynthesis of veratrole, a key volatile for pollinator attraction

**DOI:** 10.1186/1471-2229-12-158

**Published:** 2012-08-31

**Authors:** Alok K Gupta, Tariq A Akhtar, Alex Widmer, Eran Pichersky, Florian P Schiestl

**Affiliations:** 1 Institute of Systematic Botany, University of Zurich, Zurich, CH-8008, Switzerland; 2 ETH Zurich, Institute of Integrative Biology, Plant Ecological Genetics, Zurich, CH-8092, Switzerland; 3 Department of Molecular, Cellular and Developmental Biology, University of Michigan, Ann Arbor, MI, USA

**Keywords:** Floral scent, VOC, 1, 2-dimethoxybenzene, Pollination, *Hadena bicruris*

## Abstract

**Background:**

*Silene latifolia* and its pollinator, the noctuid moth *Hadena bicruris,* represent an open nursery pollination system wherein floral volatiles, especially veratrole (1, 2-dimethoxybenzene), lilac aldehydes, and phenylacetaldehyde are of key importance for floral signaling. Despite the important role of floral scent in ensuring reproductive success in *S. latifolia*, the molecular basis of scent biosynthesis in this species has not yet been investigated.

**Results:**

We isolated two full-length cDNAs from *S. latifolia* that show similarity to rose orcinol *O*-methyltransferase. Biochemical analysis showed that both *S. latifolia guaiacol O-methyltransferase1* (*SlGOMT1*) &*S. latifolia guaiacol O-methyltransferase2* (*SlGOMT2*) encode proteins that catalyze the methylation of guaiacol to form veratrole. A large K_m_ value difference between SlGOMT1 (~10 μM) and SlGOMT2 (~501 μM) resulted that SlGOMT1 is 31-fold more catalytically efficient than SlGOMT2. qRT-PCR expression analysis showed that the *SlGOMT* genes are specifically expressed in flowers and male *S. latifolia* flowers had 3- to 4-folds higher level of *GOMT* gene transcripts than female flower tissues. Two related cDNAs, *S. dioica O-methyltransferase1* (*SdOMT1*) and *S. dioica O-methyltransferase2* (*SdOMT2*), were also obtained from the sister species *Silene dioica*, but the proteins they encode did not methylate guaiacol, consistent with the lack of veratrole emission in the flowers of this species. Our evolutionary analysis uncovered that *SlGOMT1* and *SlGOMT2* genes evolved under positive selection, whereas *SdOMT1* and *SdOMT2* genes show no evidence for selection.

**Conclusions:**

Altogether, we report the identification and functional characterization of the gene, *SlGOMT1* that efficiently catalyzes veratrole formation, whereas another copy of this gene with only one amino acid difference, *SlGOMT2* was found to be less efficient for veratrole synthesis in *S. latifolia*.

## Background

White campion, *Silene latifolia* (Caryophyllaceae), emits a diverse array of volatiles to attract sphingid, geometrid, and noctuid moths for pollination [1-4]. This species also shows a pronounced day-night rhythm in odor emission, with the key compounds predominately emitted during the night [4-7]. Among commonly known pollinators for this species, *Hadena bicruris,* a noctuid moth, is a specialist nursery pollinator and obligate seed predator [8,9]. Female *H. bicruris* are not only attracted for nectaring but also for oviposition into female *S. latifolia* flowers [10]. The larvae nurture on developing seeds [11] and consume almost one fourth of the fruits developed [12,13]. Available experimental evidence indicates that the *Hadena-Silene* relationship can swing in between mutualism and antagonism [9].

Recently, the scent composition of *S. latifolia* and related species has been identified and studied for behavioral activity in the pollinators [4,10,14]. A large set of volatile compounds has been found in the *S. latifolia* floral odor bouquet [6,7,13,15] and these compounds comprise three major categories: fatty acid derivatives, aromatics, and terpenoids [6,7]. Using wind-tunnel bioassays, Dötterl et al. [13] investigated pollinators interaction with individual scent compound and uncovered that only seven (veratrole, decanal, linalool, guaiacol, phenylacetaldehyde, isopentylaldoxime, and lilac aldehydes) out of total produced compounds in *S. latifolia* flowers showed behavioral activity in *H. bicruris*. A further study based on scent composition analysis revealed that veratrole and lilac aldehydes emission is reduced four-folds after pollination, while other behaviorally active and non-active compounds remain unaltered [10]. Therefore, apart from being involved in pollinator attraction, the decrease in veratrole and lilac aldehyde emission may slow down oviposition and subsequent seed predation by *Hadena* following pollination. Phenylacetaldehyde, one of the most abundant behaviorally active compounds, is involved in floral isolation of *S. latifolia* from the closely related species *S. dioica*[6]. These investigations altogether imply that veratrole, lilac aldehydes, and phenylacetaldehyde are key odor compounds that play a central role in pollinator attraction and floral isolation [6,7,10,14]. It is presently unclear which compounds induce oviposition by *Hadena* females into female *S. latifolia* flowers. *H. bicruris* rarely oviposits into *S. dioica*[11]. However, the qualitative difference in floral volatile organic compounds (VOCs) between *S. dioica* and *S. latifolia* involves only few compounds. Veratrole, guaiacol, and benzyl benzoate are produced only in *S. latifolia* but a fatty acid derivative, nonanal is only emitted by *S. dioica*[6]. Therefore, besides quantitative scent differences [6], three compounds produced in *S. latifolia* are involved in species differentiation and presumably in maintaining the *Hadena-Silene latifolia* relationship.

During the past two decades, molecular research on *Silene* has primarily focused on sex-determination [16-19], the evolution of heteromorphic sex chromosomes [20-24], hybridization [25,26], and EST sequencing for species differentiation or marker development [27,28]. The production of copious amounts of behaviorally active volatile compounds also makes *Silene* an ideal system for investigating genes underlying volatile biosynthesis. At present, though, scent biosynthetic pathways remain uncharacterized in *Silene*. Among several scent enzymes known so far, the plant *O*-methyltransferase (OMT) family of enzymes performs a prominent role in secondary metabolism and eliminates a methyl group from S-adenosyl-L-methionine to the hydroxyl group of the substrate [29]. Besides playing a role in lignin biosynthesis [30,31], anthocyanin biosynthesis [32,33], and disease resistance [34-36], these OMTs are also involved in volatile biosynthesis [37-40]. For instance, eugenol *O*-methyltransferase (EOMT) and chavicol *O*-methyltransferase (CVOMT) methylate the substrates in order to synthesize methyleugenol and methylchavicol, respectively [41,42]. Studies in roses reported the functional characterization of *orcinol O-methyltransferases* (*OOMT1* and *OOMT2*) genes that are involved in the formation of 3-hydroxy 5-methoxytoluene and 3, 5–dimethoxytoluene (DMT), two key scent compounds of rose varieties [43,44]. Until now, several plant methyltransferases have been functionally characterized owing to their involvement in floral scent biosynthesis and flavoring properties [42,45,46].

As part of an ongoing research project to characterize key genes involved in floral scent biosynthesis in *Silene* species, we have recently developed a *S. latifolia* floral EST resource of 3,072 sequences by constructing one standard and two subtraction cDNA libraries (Gupta et al. in prep). The analysis of these sequences allowed us to characterize a wide range of candidate genes including several OMTs with high similarities to functionally characterized OMTs in other species. Here we show that two full-length coding cDNAs derived from these libraries represent *S. latifolia guaiacol O-methyltransferase1* (*SlGOMT1*) and *S. latifolia guaiacol O-methyltransferase2* (*SlGOMT2*) genes and address the following questions: 1) Do heterologously expressed proteins catalyze the formation of veratrole in *S. latifolia* and *S. dioica*? 2) How do differences in veratrole emission between day and night in *S. latifolia* controlled? 3) Are *SlGOMT* genes differentially expressed between floral and leaf tissues, and between sexes? 4) Do *SlGOMT* and *S. dioica O-methyltransferase* (*SdOMT*) genes show evidence for selection?

## Results

### Isolation and characterization of GOMT and GOMT-like cDNAs

**Figure 1:**
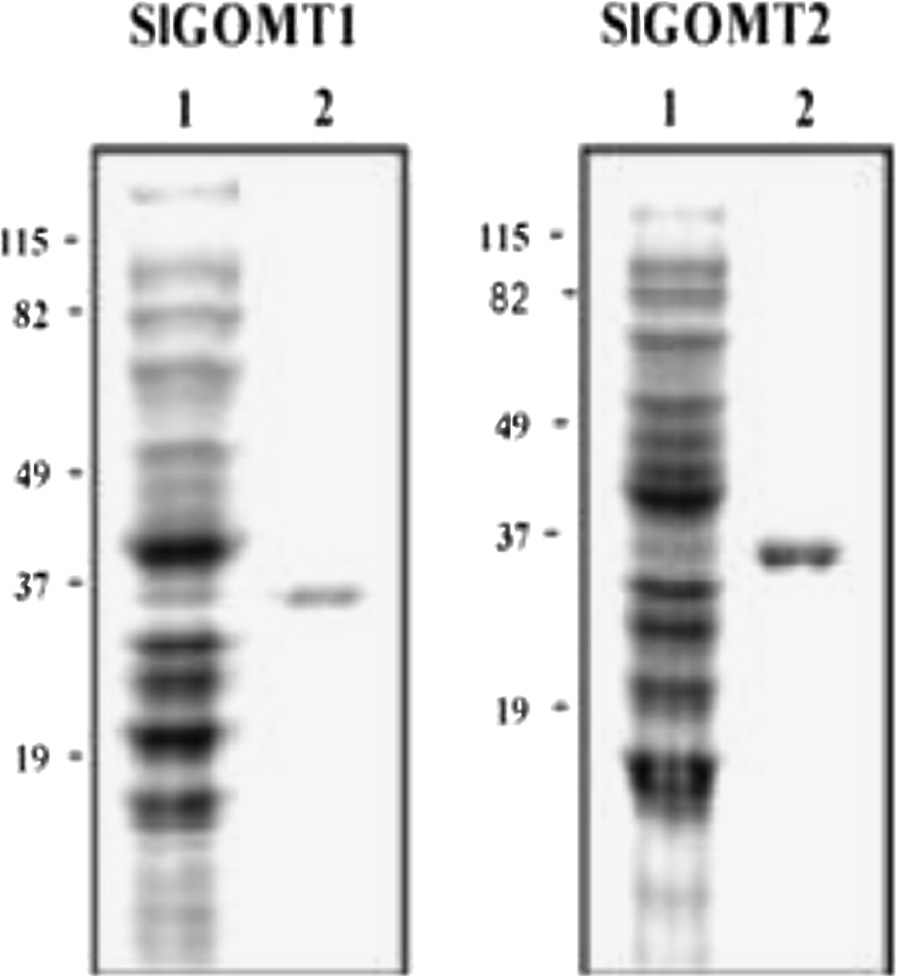
**Purification of SlGOMT1 and SlGOMT2.** Both GOMT1 and GOMT2 were purified by Ni^2+^ affinity chromatography and separated by SDS-PAGE. Lane 1 shows the soluble crude bacterial extract (~10 μg) and lane 2 shows the purified protein (~1 μg). Molecular weight markers are shown on the left.

**Figure 2:**
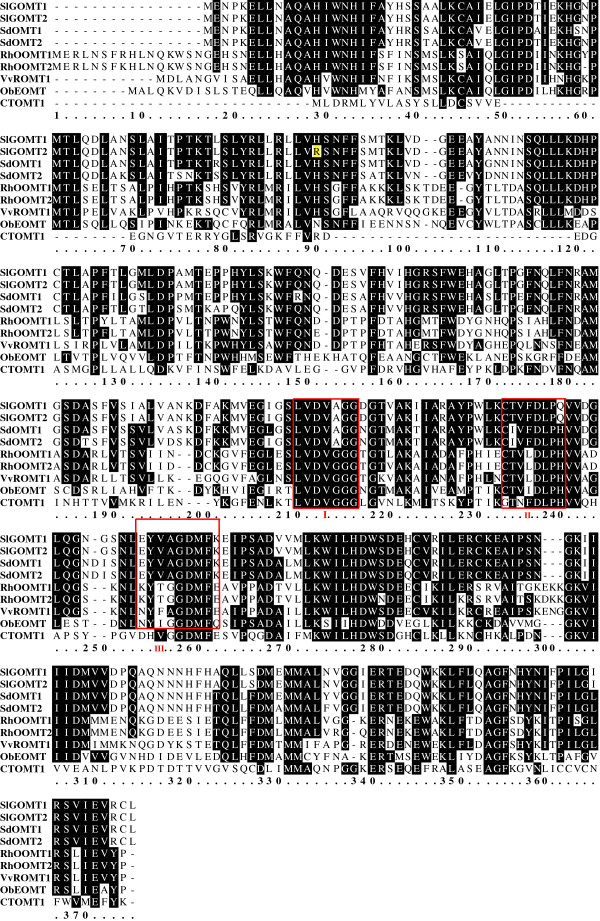
**Alignment of SlGOMT1 (*Silene latifolia*), SlGOMT2 (*Silene latifolia*), SdOMT1 (*Silene dioica*), and SdOMT2 (*Silene dioica*).** White letters on black background show identical amino acids of at least five sequences. One amino acid (in yellow background) that is involved in the difference of catalytic efficiency is highlighted*.* Three conserved SAM-binding domains are also shown in red blocks. GenBank accession numbers are as follows: RhOOMT1 (*Rosa hybrida*, NCBI: AF502433), RhOOMT2 (*Rosa hybrida*, NCBI:AF502434), VvROMT (*Vitis vinifera*, NCBI:CAQ76879), ObEOMT (*Ocimum basilicum*, NCBI:AF435008), and CTOMT1 (*Solanum lycopersicum*, sol genomics network:SGN-U582403).

A search of the EST database constructed from *S. latifolia* flowers (Gupta et al. in prep) for sequences homologous to known *O*-methyltransferases identified ESTs, and the sequence information in these ESTs led to the isolation of two coding cDNA sequences of 1,059 bp that we designated as SlGOMT1 and SlGOMT2. These sequences encode peptide sequences of 353 amino acids and the molecular mass of both purified SlGOMT proteins was approximately 37 kD on SDS-PAGE, similar to other plant-based methyltransferases [29] (Figures 1). SlGOMT1 and SlGOMT2 were nearly identical with the exception of two nucleotides differences that result in one amino acid difference at position 74. This weak divergence suggests that the two sequences correspond to different alleles of the same locus. An alignment of the deduced SlGOMT1 and SlGOMT2 protein sequences with other characterized protein sequences is shown in Figure 2. The comparison of deduced amino acid sequences between SlGOMTs (SlGOMT1 and SlGOMT2) and biochemically characterized OMTs of other plants revealed the 54–55% identity with orcinol OMTs from *Rosa hybrida*[43] and 54% identity with resveratrol OMT from *Vitis*[35]. Further Blast searches also showed 45% identity with *Ocimum* eugenol OMT [42] and 31% identity with *Solanum* catechol OMT [47]. Based on primers designated for SlGOMT1 and SlGOMT2, we were also able to obtain two GOMT-like coding cDNAs of 1,062 bp long from RNA extracted from *S. dioica* flowers and we designated them SdOMT1 and SdOMT2. These both sequences shared 89–90% identity with SlGOMT1 and SlGOMT2. 

### Biochemical characterization of SlGOMTs and SdOMTs

**Figure 3:**
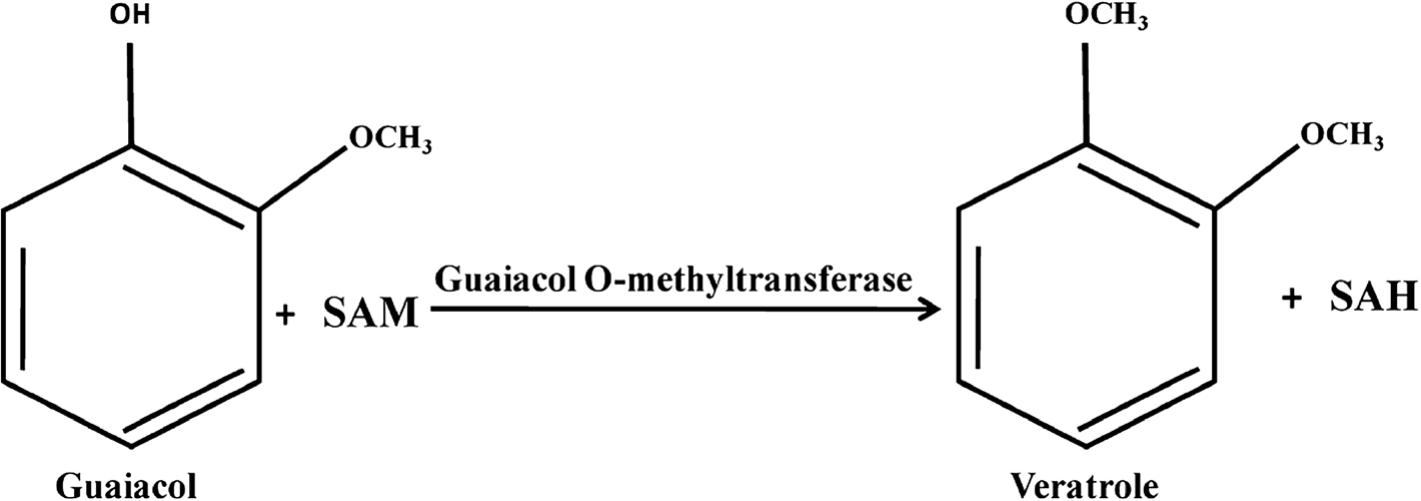
**Biosynthesis of veratrole in*Silene latifolia.*** A methyl group from S-adenosyl-L-methionine (SAM) is transferred to the p-hydroxyl group of guaiacol to synthesize veratrole and S-adenosyl-L- homocysteine (SAH).

**Figure 4:**
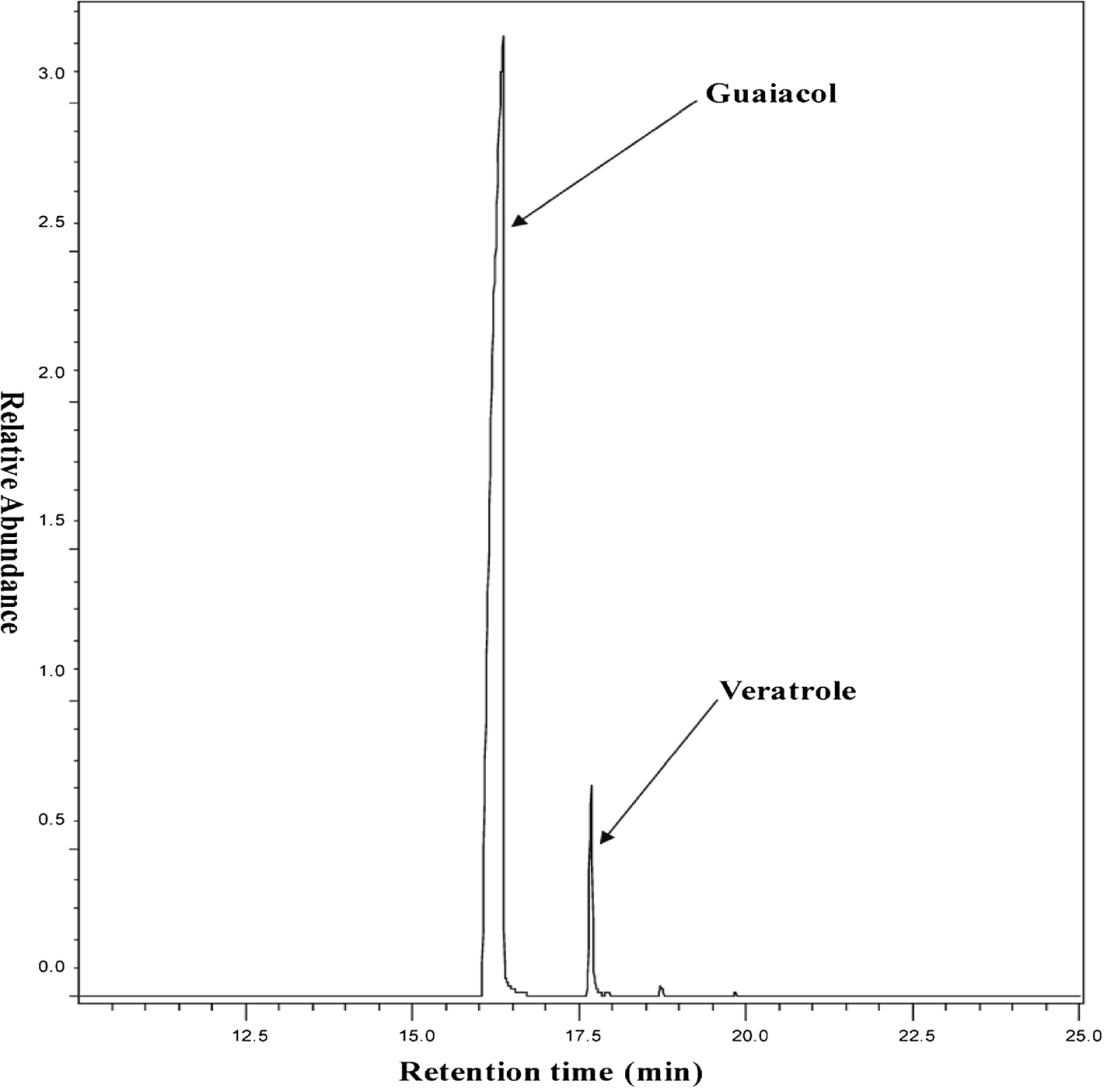
**A representative GC chromatogram showing the conversion of guaiacol to veratrole by SlGOMT1.** A desalted crude extract from *E. coli* cells expressing SlGOMT1 was supplied with guaiacol and SAM. Volatile compounds were collected and analyzed as described in methods section.

**Figure 5:**
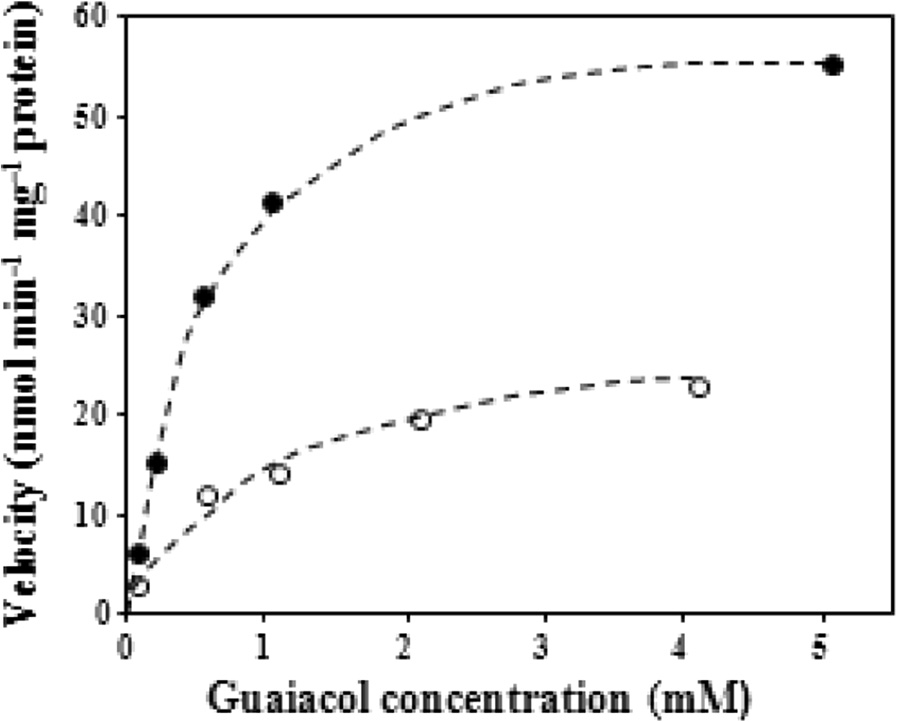
Velocity versus substrate curves for SlGOMT1 (closed circles) and SlGOMT2 (open circles) with increasing amounts of guaiacol.

SlGOMTs and SdOMTs were expressed in *E. coli* and the proteins tested for methylation activity with guaiacol, the presumed substrate of veratrole, as well as orcinol, the substrate of OOMT, eugenol, the substrate of EOMT, and catechol, a compound recently shown to be the substrate of a methyltransferase in tomato, which converts it to guaiacol [47]. Methyleugenol, which has no hydroxyl groups that could be methylated, was used as a control (Table 1). SlGOMT1 exhibited preferred activity with guaiacol and was efficiently able to methylate guaiacol to veratrole (Figures 3, 4, 5 & Additional file 1: Figure S4), with a K_m_ value for guaiacol of 9.8 μM (Table 2). SlGOMT2 had low levels of activity with several substrates, including guaiacol (Table 1) and a K_m_ value for guaiacol, 501 μM, that is 51-fold higher than that of SlGOMT1 (Table 2), resulting an enzyme that is 31-fold less efficient with guaiacol than SlGOMT1 (Table 2). SdOMT1 and SdOMT2 did not methylate any of these tested substrates. 

**Table 1:** Substrate specificity of SlGOMT1 and SlGOMT2 with various substrates

**Substrate**	SlGOMT1* (% Activity)	**SlGOMT2 (% Activity)**
Guaiacol	100	12
Catechol	35	13
Orcinol	11	9
Eugenol	<1	<1
Methyleugenol	<1	<1

Purified SlGOMT1 and SlGOMT2 were assayed at room temperature under standard assay conditions by adding substrate at a final concentration of 200 μM and excess ^14^C-SAM together. Relative activity is expressed as the percentage of GOMT1 activity with guaiacol as a substrate.

* Specific activity was measured 1.96 NMOL/MIN/MG protein at saturating substrate concentrations.

**Table 2:** Kinetic parameters of SlGOMT1 and SlGOMT2

**Recombinant protein**	***K***_**m**_	***K***_**cat**_	***K***_**cat**_**/*K***_**m**_
	***μM***	***s***^***-1***^	***s***^***-1***^ ***M***^***-1***^
SlGOMT1	9.79 ± 1.51	2.58 × 10^-3^ ± 1.64× 10^-4^	270.27 ± 64.10
SlGOMT2	501 ± 198	3.95 × 10^-3^ ± 1.09× 10^-3^	8.63 ± 1.81

### Veratrole emission

Veratrole emission from both female and male flowers was 50–77 orders of magnitude higher during the night than during the day (Z = −3.321, *p* = 0.001; Table 3). As expected, there was no veratrole emission detected from leaf tissue, indicating that veratrole is a flower-specific compound (leaf volatile data is not shown in Table 3). However, there was no significant difference in veratrole emission for male and female flowers (Z = 0, *p* = 1).

**Table 3:** **Veratrole emission and SlGOMT expression in*Silene latifolia*flowers**

**Flower / Condition**	**Expression (Mean ± SE)**	**Veratrole emission**^**1**^**(Mean ± SE)**
Night flowering^2^ ♀	0.31 ± 0.08	1.36 ± 0.49
Day flowering^2^ ♀	0.43 ± 0.08	0.027 ± 0.027
Night flowering^2^ ♂	1.11 ± 0.28	3.86 ± 3.17
Day flowering^2^ ♂	1.24 ± 0.20	0.05 ± 0.038

^1^ ng/l, ^2^ n = 5.

### Gene expression analysis

To ascertain whether *SlGOMT* genes are differentially expressed between flowers and leaves in *S. latifolia*, qRT-PCR analyses were performed (Table 3). Our results show that *SlGOMT* genes are preferentially expressed in floral tissue, whereas no expression was detected in leaf tissues (Table 3, data is not shown for leaf tissue in this table). Surprisingly, there was no significant difference found in *SlGOMT* gene expression in flowers collected during day and night (Z = −0.680, *p* = 0.529). However, *SlGOMT* expression is significantly (3–4 folds) higher in males than in females during day and night (Z = −3.250, *p* = 0.001).

### Evolutionary analysis

**Figure 6:**
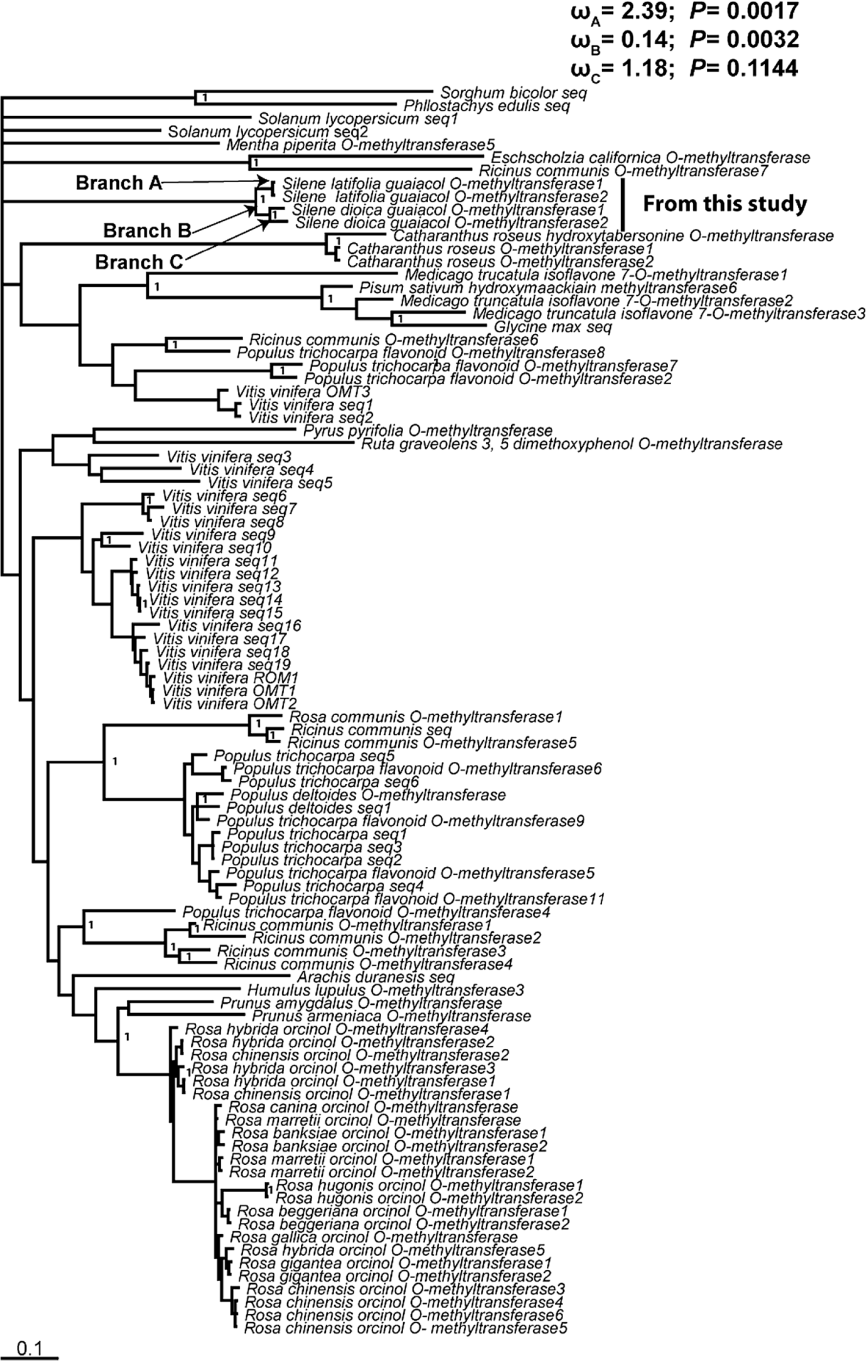
**Phylogenetic analysis of*Silene*GOMT homologs.** Bayesian phylogeny with branch length from BaseML is shown and numbers indicate posterior probabilities (only 1.0 value mentioned) next to branches.

To analyze evolutionary relationships and patterns of sequence evolution in *SlGOMTs* and *SdOMTs* sequences, we retrieved a total of 92 plant OMT sequences from NCBI (Additional file 2: Table S1) following the criteria described in the methods section. These sequences were combined with our *SlGOMTs* and *SdOMTs* sequences to construct a Bayesian inference phylogeny. All identified *Silene* sequences formed a separate clade and showed distant relationships with *M. truncatula isoflavone7-O-methyltransferase* (*IOMT*) gene. A maximum likelihood-based analysis of synonymous versus non-synonymous mutations was performed to test for the signature of selection. Our analysis revealed strong evidence for positive selection (ω = 2.38; *p* = 0.0017) along the branch leading to *SlGOMT1* and *SlGOMT2*, whereas no evidence for positive selection was found along the branch to *SdOMT1* and *SdOMT2* (Additional file 3: Table S2). Purifying selection was found along the branch linking the *SlGOMT* and *SdOMT* sequences (ω =0.14; *p* = 0.0032; Figure 6).

## Discussion

In this paper, we report the identification of a novel enzyme that is responsible for the formation of veratrole (1, 2-dimethoxybenzene) from guaiacol in *Silene latifolia*. Veratrole is widely found among plants and is known as a key compound for some plant-insect interactions. In *S. latifolia* and other *Silene* species, veratrole is a key compound for pollinator attraction [5,13,48]. The related 1, 4-dimethoxybenzene volatile compound has been identified from the flowers of *Salix* species and this compound serves as an attractant for an oligolectic bee [49]. Schiestl & Dötterl [50] have also recently shown the evolutionary importance of methoxlated aromatics including veratrole in the association of Araceae plants and their pollinators, but until our study, the molecular basis of veratrole synthesis was unknown in planta.

### GOMT and veratrole biosynthesis

The dioecious *Silene latifolia* serves as a plant model system for several ecological and evolutionary topics due to its remarkable features including well differentiated heteromorphic sex chromosomes, its nursery pollination system, and the associated floral fragrances [6,7,13,23,51-55]. In *S. latifolia*, aromatic (e. g., veratrole, phenylacetaldehyde, methylsalicylate, and benzyl benzoate) and montoterpenoid (e. g., lilac aldehydes & alcohol, α-pinene, and linalool) compounds comprise a significant part of the total floral scent emission [6,7]. Genes involved in the synthesis of these few widespread floral volatiles have been characterized in a number of plants, mostly from hermaphroditic species. For instance, genes that are involved in the synthesis of phenylacetaldehyde have been characterized in *Petunia*[56], *Rosa*[57], *Lycopersicum*[58], and *Arabidopsis*[59]. Similarly, a large number of *terpene synthase* genes have been isolated and characterized in several plant species [60-66]. However, few scent genes have been examined in dioecious species, with one exception being the two *terpene synthase* genes that are accountable for the synthesis of some sesquiterpenes in *Actinia deliciosa* flowers [67]. One of these two terpene synthase genes was found to possess conifer diterpene internal sequence (CDIS) domain that is usually characteristic feature of many diterpene synthase and determines their activity [67,68].

Veratrole is a key attractant compound in the floral fragrance of *S. latifolia* and it is down-regulated, together with other compounds, subsequent to pollination [10]. Our study in this dioecious plant species characterized two candidate "veratrole forming genes", *SlGOMT1* and *SlGOMT2*. Analysis of the activities of the enzymes they encode showed that SlGOMT1 is an efficient and specific guaiacol methyltransferase (Tables 1, 2, and Figures 3,5) whose activity leads to the synthesis of veratrole. SlGOMT1 also had low levels of activity (<11% compared with its activity with guaiacol) with several other substrates, with the exception of catechol (Table 2). SlGOMT2, on the other hand, has low levels of activity with guaiacol compared to the activity of SlGOMT1 (Tables 1, 2, and Figure 5), and comparably low levels with several other substrates tested. Therefore, it is not possible to conclude at this point that the presence of SlGOMT2 in the flowers would lead to the synthesis of veratrole, particularly since the K_m_ value of SlGOMT2 is relatively high compared with the corresponding value of SlGOMT1 (app. 500 μM vs.10 μM, Table 2). An examination of the internal concentration of guaiacol in flower tissue is required to resolve this issue.

It has recently been reported that catechol is the substrate of a methyltransferase in tomato fruit (CTOMT) that converts it to guaiacol [47]. Thus, catechol may serve as a precursor of guaiacol in *S. latifolia* as well. Although SlGOMT1 does show a relatively low level of amino acid similarity to tomato CTOMT, we observed a moderate level of activity for SlGOMT1 with catechol (Table 1). While this result is intriguing, additional work such as measuring catechol concentrations in *S. latifolia* flowers must be carried out to determine whether catechol is indeed the precursor of guaiacol in this system. In tomato, both catechol and guaiacol have been detected in the fruit, but in *S. latifolia* flowers, only guaiacol emission, but not catechol, have been observed [6,7,10,47].

It is well established that in many species, flower scent emission is governed by a circadian clock and/or light, apparently as an adaption to the temporal activity of their pollinators [37,38,69-71]. Our study and that of Wälti et al. [6] show that scent emission in *S. latifolia* differs between day and night. Furthermore, *SlGOMT* expression in male flowers was 3–4 folds higher than in female flowers both during the day and night but this difference was not found for sex-specific veratrole emission. Wälti et al. [7] found significant higher veratrole emission in male compared to female *S. latifolia* plants and the lack of a significant sex specific difference in our study may be consequence of relatively low sample size combined with high variability in veratrole emission. It is worth noting, however, that Nieuwenhuizen et al. [67] found that *A. deliciosa* female flowers produce more scent than male flowers even though no difference in gene expression was found between flowers of the two sexes.

### Evolution of GOMT

The observation that a single amino difference between SlGOMT1 and SlGOMT2 is responsible for a large difference in catalytic efficiency of veratrole synthesis in *S. latifolia* is intriguing. Similarly, two duplicated gene copies were characterized for eugenol synthesis in *Clarkia breweri* in which one copy is about 3.5 fold more efficient than the other [72].

In contrast to *S. latifolia* which emits veratrole in its floral odor, *S. dioica* emits no veratrole [6]. The two genes found in that species that are closely related to *SlGOMTs*, *SdOMT1*, and *SdOMT2*, were found to be expressed in floral tissue, but, our data show that *SdOMT1* and *SdOMT2* do not have activity with guaiacol, catechol, or the other substrates tested in this study. It is possible that these enzymes contain mutations that render them inactive. Koeduka et al. [73] found that a single nucleotide substitution in the coding region of the *Petunia axillaris subsp parodii isoeugenol synthase* (*PapIGS*) gene has resulted to the loss of the enzymatic activity and thereby, prevents isoeugenol emission from flowers. On the other hand, several studies on the plant OMT family have revealed that one or a few amino acid changes can be responsible for novel substrate specificities, such as in the *Clarkia* iso(eugenol) OMT [45] and *Ocimum* phenylpropene-OMTs [74]. Such amino acid changes in OMT sequences have been found to evolve under positive selection [75,76].

Because the sequences of *SlGOMT1* and *SlGOMT2* are more similar to each other than to *SdOMT1* and *SdOMT2* (Figure 6), we do not know if they represent two alleles of the same locus, or a duplication that occurred after the split from *S. dioica*. The results of the sequence divergence analysis indicate positive selection on *SlGOMT1* &*SlGOMT2 (*as evidenced by their significant dN/dS value, 2.39)*,* suggesting the recent acquisition of GOMT activity in *S*. *latifolia* by a few amino acid substitutions. This evolutionary change could be brought about through selection for better discrimination by pollinators from its closely related sister species, *S. dioica*, leading to enhanced floral isolation [77,78]. Alternatively, veratrole could be a key signal in the specific association of *S. latifolia* with its primary pollinator, *Hadena bicruris*. Pollinator-mediated selection is commonly occurring on scent compounds [79,80]. Higher amount of veratrole emission can empower higher flower attractiveness from longer distances in *S. latifolia*[7,13,81]. Although there is no selection detected on *SdOMTs*, the evidence for purifying selection before in the branch leading to both *SlGOMTs* and *SdOMTs* suggest a conserved role of the ancestral OMT proteins, and it is therefore possible that the SdOMT enzymes have another, as yet uncharacterized functions.

## Conclusions

Two novel flower-specific methyltransferase genes were characterized from *S. latifolia* that are capable of methylating guaiacol and is expressed differentially between male and female individuals. Our study also provides the foundation for future *Silene* scent molecular research. Altogether, the information provided on genes responsible for scent production and its evolutionary signatures will be relevant for understanding pollinator-driven selection on floral scent variation in plants.

## Methods

### Plant materials

*S. latifolia* and *S. dioica* plants were grown under green house conditions at the Hönggerberg and Eschikon experimental sites of ETH Zurich. Flowers were harvested after dusk (mid night). The flowers were snap-frozen in liquid nitrogen after collection and stored at −80°C until use. Total RNAs were extracted from flowers using the RNeasy plant mini kit (Qiagen) and poly A + mRNA was isolated using the Oligotex mRNA kit (Qiagen) according to the manufacturer's instructions.

### Isolation of full-length SlGOMTs & SdOMTs

*S. latifolia* floral EST libraries were searched against sequences with homology to known members of plant methyltransferase families using the BLASTX algorithm [82]. One partial sequence showing similarity with rose *orcinol O-methyltransferase* was selected for isolation of the full-length cDNA sequence. Complete coding cDNA sequences were isolated using the SMART-RACE cDNA amplification kit (Clontech) according to the manufacturer's protocol. Gene-specific primer pairs were used to obtain the full-length cDNA sequence from *S*. *latofolia* as well as from *S. dioica*, and the resulting PCR products were cloned into the pSMART vector (Lucigen). Inserts were verified by sequencing. Two coding sequences each from *S. latifolia* and *S. dioica* were isolated.

### Preparation of *Silene* GOMT constructs

To determine whether *SlGOMT1*, *SlGOMT2*, *SdOMT1*, and *SdOMT2* potentially encode functional proteins, we amplified the complete open reading frames including start codons but without native stop codons at the end. The protein coding regions (ORFs) of *SlGOMT*s and *SdOMT*s were amplified by RT-PCR using an upstream primer and a downstream primer (Additional file 4: Table S3). PCR amplification was carried out for 30 cycles after an initial denaturation at 94°C for 2 min. Each cycle consisted of denaturation at 94°C for 1 min, annealing at 55°C for 1 min and extension at 72°C for 2 min with a final extension of 7 min in a Biometra thermocycler. The resulting PCR products were cloned into expression vector using pEXP-CT/Topo TA expression kit according to manufacturer's instructions. The constructs were subjected to complete sequencing to confirm the orientation of the inserts.

### GOMT activity, purification, enzymatic assays, and product identification

*SlGOMT1*, *SlGOMT2, SdOMT1,* and *SdOMT2* constructs were transformed into *E. coli* BL21-CodonPlus (DE3)-RIPL cells (Stratagene). Cells harboring constructs were cultured in LB medium until an OD_600_ of 0.6. Isopropyl 1-thio-β-D-galactopyranoside (IPTG) was added to a final concentration of 1 mM to induce protein expression and cells were incubated for 16 hours at 18°C. Cell pellets resuspended in Buffer A (100 mM Tris–HCl, 5 mM MgCl_2_, 10 mM β-mercaptoethanol, 10% glycerol [v/v], pH 7.5) were subsequently ruptured by sonication. Clarified crude extracts were desalted on PD-10 columns equilibrated with Buffer A and protein concentration was estimated with the standard Bradford method. Enzymatic activity was performed essentially as described by Wang et al. [83]. Enzyme (10–20 μg of desalted crude extract) was incubated in a final volume of 50 μl with 0.2 mM substrate (guaiacol, orcinol, catechol, eugenol, and methyleugenol) and 5 μM S-[methyl-^14^C]adenosyl-l-Met (40–60 mCi/mmol) for 30 minutes at room temperature. Reaction products were extracted with 200 μl of ethyl acetate and 100 μL of the organic phase was transferred to 2 ml of non-aqueous scintillation fluid and subjected to a scintillation counter (model 2S6800, Beckman). Approximately 50 μg of desalted crude extract was incubated in a final volume of 200 μl containing 1 mM guaiacol and 1 mM SAM in buffer A for 30 minutes at room temperature and SPME device was employed for volatile collection.

The recombinant SlGOMT1 and SlGOMT2 proteins produced in *E. coli* were purified by Ni^2+^ affinity chromatography (Qiagen), according to the manufacturer’s instructions. Briefly, crude protein extracts were desalted into Buffer B (100 mM potassium phosphate, 150 mM NaCl, 10 mM imidazole, pH 7.5) and applied to 0.5 mL of Ni^2+^ resin. Following 10 column washes with Buffer B, proteins bound to the resin were eluted with Buffer B containing 250 mM imidazole. Eluted proteins were desalted into Buffer A and concentrated using Amicon ultra-15 filters (Millipore). Protein purity of the eluted proteins was assessed by SDS-PAGE and concentration was determined as described above. For kinetic analysis, assays were performed as described above with ~0.5 μg of purified SlGOMT1 or 2 and various concentrations of guaiacol, while SAM concentration was held constant at 200 μM. Reactions were stopped by the addition of 10 μL of 2 N HCL. Double reciprocal plots were used to determine the apparent K_m_ and V_max_ for each enzyme.

### Scent collection

Floral odor was collected in a climate chamber using the dynamic heaspace method. We collected odor at light from 4 AM to 8 PM and in the dark from 9 PM to 3 AM. Five individuals each of both sexes were used for floral volatile collection and three individuals of each sex were selected for leaf volatile collection. Intact newly opened whole flowers and new leaves of these individuals were enclosed in an oven roasting bag (Nalophan®) and air was sucked out from these bags using a battery operated pump (PAS-500 Personal Air Sampler, Spectrey, Redwood city, California, USA). Volatiles were trapped in self made absorbent glass tubes filled with Tenax for 30 min during day and night. Control samples were also collected for discriminating the compounds from surrounding air contaminations and their average was subtracted from the each sample. All glass filters were capped with teflon tape on both sides and enclosed in the aluminium foil to avoid contamination, and samples were preserved for subsequent gas chromatograph (GC) analysis at −20°C.

### Quantitative gas chromatographic analysis and compound identification

Volatile samples were analyzed within 4 days of volatile collections by a gas chromatograph (GC, Agilent 6890 N) connected to an HP5 column (30 m × 0.32 mm internal diameter × 0.25 μm film thickness) and an Agilent 5975 mass selective detector associated to a thermal desorption system (TDS2, Gerstel, Mühlheim Germany). The TDS temperature was adjusted to rise from 30° (0.5-min hold) to 240°C (1-min hold) at 60°C per minute. The CIS (cold injection system) temperature was set to rise from −50°C (0.5-min hold) to 150°C (0.5-min hold) at 16°C per second and to 250°C (0.5-min hold) at 12°C per second. The oven temperature of the GC6890 was programmed to rise from 50°C (3 min hold) to 230°C at 8°C per minute. Agilent ChemStation and MSD ChemStation E.02.00.493 (Agilent Technologies, Palo Alto, California, USA) software were utilized for characterizing the peaks and retention times. Synthetic veratrole (Sigma–Aldrich**,** Switzerland) was further employed as a standard for comparing the retention time as well as calibrating peak area with absolute amount in the mass spectrometry chromatograms. Quantified amount of veratrole was estimated in ng per liter of air sampled.

### Real time RT-PCR analysis

We used the same set of flower and leaf samples for gene expression analysis that were also used for volatile collections. After each volatile collection, floral and leaf tissues were immediately harvested and snap-frozen in liquid nitrogen. All these samples were stored at −80°C until required. Total RNA was extracted from flowers and leaf using the RNeasy plant mini kit (Qiagen). RNA was subsequently subjected to DNAse treatment using RQ1 RNase-Free DNase (Promega, Madison, Wisconsin, USA) following manufacturer’s instructions. First-strand cDNA was synthesized by M-MLV Reverse Transcriptase (Promega, Madison, Wisconsin, USA) and an Oligo(dT )_15_ primer. As a control for possible DNA contamination, reverse transcriptions were done with and without the enzyme. RT-PCRs were performed with the SYBR Master Mix (Applied Biosystems, Foster City, USA) on a 7500 Real Time PCR System (Applied Biosystems, Foster City, USA). Actin, EF1A, and CL285 primers were applied as internal controls and qRT-GOMT primers were used for amplification (Additional file 4: Table S3). All samples including control reactions were performed three times. The results were recorded by 7500 Software v 2.0.1 (Applied Biosystems, Foster City, USA). At least three sets of independent experiments were performed to calculate a mean cycle threshold (Ct) value and standard deviations.

### Statistical analysis

Non-parametric Mann Whitney U-tests were used to analyze the differences in veratrole emission and gene expression between sexes and day/night. All analyses were performed using SPSS statistical package (SPSS Inc. Chicago).

### Phylogeny reconstruction and selection analysis

SlGOMT1 sequence was chosen as a seed for retrieval of homologous sequences from NCBI using BlastN searches and combined with the sequences that were generated from this study. BlastN searches were optimized using ‘somewhat similar sequences’ option following general parameters (Max target sequences 100, expect threshold 10, word size 11, and match/mismatch scores 2,-3). DNA sequences were aligned with BioEdit v7.0.5 and poorly aligned 8 sequences from the pool of selected 100 sequences were excluded for subsequent analysis. The best substitution model was estimated using Mr. Modeltest 2.3 [84]. The Markov Chain Monte Carlo (MCMC) method was employed to approximate the posterior probabilities of trees using MrBayes 3.1.2 [85]. Branch lengths of obtained consensus tree were optimized with BaseML. CodeML was further used for a codon-based model. Both programs belong to PAML 4.4 package [86].

## Competing interest

The authors declare that they have no competing interest.

## Authors' contributions

AKG, FPS, and EP conceived the project. AKG carried out experiments, analyses, and wrote the manuscript. TA contributed to SlGOMTs biochemical assays & protein purification. FPS, EP, and AW assisted in experiment design and contributed to manuscript preparation. All authors read the manuscript and approved it.

## Supplementary Material

Additional file 1 **Figure S4.** Comparison of mass spectra of veratrole. Click here for file

Additional file 2 **Table S1.** Sequences used for phylogenetic analysis. Click here for file

Additional file 3 **Table S2.** Analysis of selection using PAML. Click here for file

Additional file 4 **Table S3.** Primers used in this study. Click here for file
