# Supplementary figures and images for: Identification of white campion (Silene latifolia) guaiacol O-methyltransferase involved in the biosynthesis of veratrole, a key volatile for pollinator attraction
Source: BMC Plant Biol. 2012 Aug 31;12:158. doi: 10.1186/1471-2229-12-158 (PMC3492160; doi:10.1186/1471-2229-12-158)

**(a) Mass spectra of product formed by SIGOMT1**

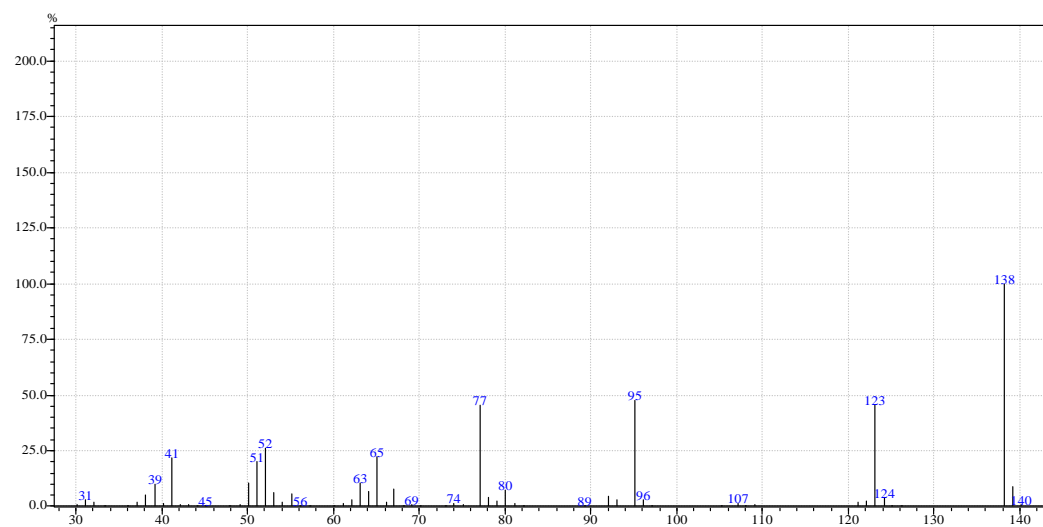

**(b) Mass spectra of veratrole authentic standard**

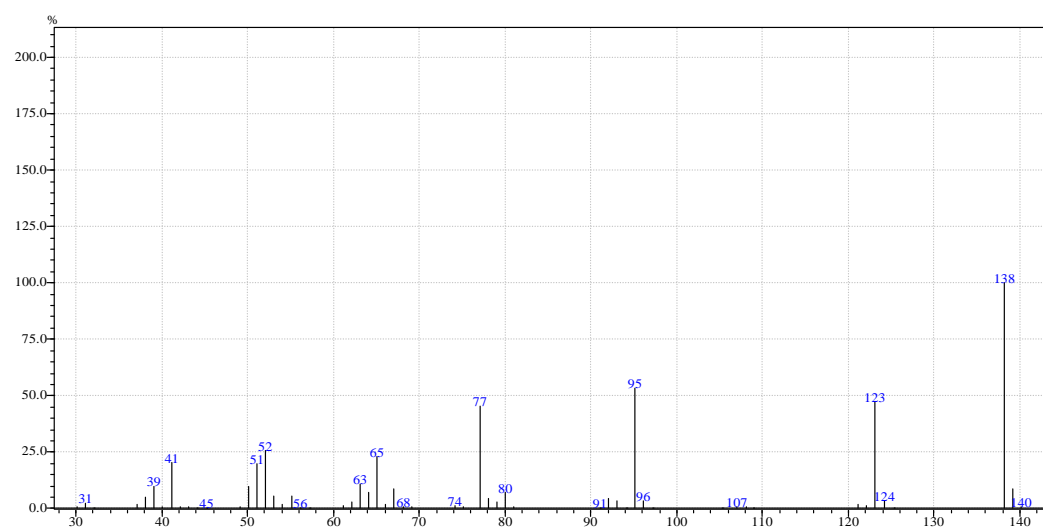

Supplement: Additional file 1 — Figure S4. Comparison of mass spectra of veratrole. [file 1471-2229-12-158-S1.pdf]
