# Supplementary material for: Identification of white campion (Silene latifolia) guaiacol O-methyltransferase involved in the biosynthesis of veratrole, a key volatile for pollinator attraction
Source: BMC Plant Biol. 2012 Aug 31;12:158. doi: 10.1186/1471-2229-12-158 (PMC3492160; doi:10.1186/1471-2229-12-158)
Supplement: Additional file 2 — Table S1. Sequences used for phylogenetic analysis. [file 1471-2229-12-158-S2.pdf]

1	XM_002461644.1	*Sorghum bicolor* seq
2	FP096422.1	*Phllostachys edulis seq*
3	AK247737.1	*Solanum lycopersicum seq1*
4	AK320249.1	*Solanum lycopersicum* seq2
5	AY337462.1	*Mentha piperita O- methyltransferase5*
6	EU882970.1	*Eschscholzia californica O-methyltransferase*
7	XM_002517911.1	*Ricinus communis O-methyltransferase7*
8	SlGOMT1 (From this study)	*Silene latifolia guaiacol O-methyltransferase1*
9	SlGOMT2 (From this study)	*Silene latifolia guaiacol O-methyltransferase2*
10	SdOMT1 (From this study)	*Silene dioica guaiacol O-methyltransferase1*
11	SdOMT2 (From this study)	*Silene dioica guaiacol O-methyltransferase2*
12	EF444545.1	*Catharanthus roseus hydroxytabersonine O-methyltransferase*
13	EF444544.1	*Catharanthus roseus O-methyltransferase1*
14	EF444546.1	*Catharanthus roseus O-methyltransferase2*
15	AY942159.1	*Medicago trucatula isoflavone 7-O-methyltransferase1*
16	U69554.1	*Pisum sativum hydroxymaackiain methyltransferase6*
17	AY942158.1	*Medicago truncatula isoflavone 7-O-methyltransferase2*
18	DQ419913.1	*Medicago truncatula isoflavone 7-O-methyltransferase3*
19	BT096188.1	*Glycine max seq*
20	XM_002522455.1	*Ricinus communis O-methyltransferase6*
21	XM_002331924.1	*Populus trichocarpa flavonoid O-methyltransferase8*
22	XM_002331923.1	*Populus trichocarpa flavonoid O-methyltransferase7*
23	XM_002305068.1	*Populus trichocarpa flavonoid O-methyltransferase2*
24	EF535147.1	*Vitis vinifera OMT3*
25	XM_002278172.1	*Vitis vinifera seq1*
26	XM_002278109.1	*Vitis vinifera seq2*
27	AB014456.1	*Pyrus pyrifolia O-methyltransferase*
28	AY894417.1	*Ruta graveolens 3 5 dimethoxyphenol O-methyltransferase*
29	XM_002278113.1	*Vitis vinifera seq 3*
30	XM_002278070.1	*Vitis vinifera seq 4*
31	XM_002262797.1	*Vitis vinifera seq 5*
32	XM_002278362.1	*Vitis vinifera seq 6*
33	XM_002278280.1	*Vitis vinifera seq 7*
34	XM_002278481.1	*Vitis vinifera seq 8*
35	XM_002278154.1	*Vitis vinifera seq 9*
36	XM_002278175.1	*Vitis vinifera seq 10*
37	XM_002278021.1	*Vitis vinifera seq 11*
38	XM_002273983.1	*Vitis vinifera seq 12*
39	XM_002274033.1	*Vitis vinifera seq 13*
40	XM_002277872.1	*Vitis vinifera seq 14*
41	XM_002277891.1	*Vitis vinifera seq 15*
42	XR_077883.1	*Vitis vinifera seq 16*
43	XM_002278258.1	*Vitis vinifera seq 17*

44	XR_077985.1	*Vitis vinifera seq 18*
45	XM_002281332.1	*Vitis vinifera seq 19*
46	FM178870.1	*Vitis vinifera ROMT1*
47	XM_002281445.1	*Vitis vinifera OMT1*
48	EF535146.2	*Vitis vinifera OMT2*
49	XM_002517786.1	*Rosa communis O-methyltransferase1*
50	XM_002517789.1	*Ricinus communis seq*
51	XM_002517787.1	*Ricinus communis O-methyltransferase5*
52	XM_002338064.1	*Populus trichocarpa seq5*
53	XM_002330641.1	*Populus trichocarpa flavonoid O-methyltransferase6*
54	XM_002335396.1	*Populus trichocarpa seq6*
55	AJ698925.1	*Populus deltoides O*-methyltransferase
56	EF148749.1	*Populus deltoides seq1*
57	XM_002332494.1	*Populus trichocarpa flavonoid O-methyltransferase9*
58	XM_002332497.1	*Populus trichocarpa seq1*
59	XM_002334049.1	*Populus trichocarpa seq3*
60	XM_002332498.1	*Populus trichocarpa seq2*
61	XM_002327160.1	*Populus trichocarpa flavonoid O-methyltransferase5*
62	XM_002327161.1	*Populus trichocarpa seq4*
63	XM_002334766.1	*Populus trichocarpa flavonoid O-methyltransferase11*
64	XM_002326000.1	*Populus trichocarpa flavonoid O-methyltransferase4*
65	XM_002517781.1	*Ricinus communis O-methyltransferase1*
66	XM_002538856.1	*Ricinus communis O-methyltransferase2*
67	XM_002525113.1	*Ricinus communis O-methyltransferase3*
68	XM_002525114.1	*Ricinus communis O- methyltransferase4*
69	HP001058.1	*Arachis duranesis seq*
70	EU309727.1	*Humulus lupulus O -methyltransferase3*
71	AJ223151.1	*Prunus amygdalus O- methyltransferase*
72	U82011.1	*Prunus armeniaca O-methyltransferase*
73	AJ439744.1	*Rosa hybrida orcinol O-methyltransferase4*
74	AF502434.1	*Rosa hybrida orcinol O-methyltransferase2*
75	AJ439742.1	*Rosa chinensis orcinol O-methyltransferase2*
76	AJ439743.1	*Rosa hybrida orcinol O-methyltransferase3*
77	AF502433.1	*Rosa hybrida orcinol O-methyltransferase1*
78	AJ439741.1	*Rosa chinensis orcinol O-methyltransferase1*
79	AM182837.1	*Rosa canina orcinol O-methyltransferase*
80	AM182809.1	*Rosa marretii orcinol O-methyltransferase*
81	AM182769.1	*Rosa banksiae orcinol O-methyltransferase1*
82	AM182770.1	*Rosa banksiae orcinol O-methyltransferase2*
83	AM182810.1	*Rosa marretii orcinol O-methyltransferase1*
84	AM182848.1	*Rosa marretii orcinol O- methyltransferase2*
85	AM182806.1	*Rosa hugonis orcinol O-methyltransferase1*
86	AM182805.1	*Rosa hugonis orcinol O-methyltransferase2*

87	AM182774.1	*Rosa beggeriana orcinol O-methyltransferase1*
88	AM182771.1	*Rosa beggeriana orcinol O-methyltransferase2*
89	AM182797.1	*Rosa gigantea orcinol O-methyltransferase1*
90	AM182795.1	*Rosa gigantea orcinol O-methyltransferase2*
91	AM182792.1	*Rosa gallica orcinol O-methyltransferase*
92	AJ786311.1	*Rosa hybrida orcinol O-methyltransferase5*
93	AM182786.1	*Rosa chinensis orcinol O-methyltransferase3*
94	AM182780.1	*Rosa chinensis orcinol O-methyltransferase4*
95	AM182787.1	*Rosa chinensis orcinol O-methyltransferase6*
96	AM182785.1	*Rosa chinensis orcinol O-methyltransferase5*
