# Supplementary material for: Identification of white campion (Silene latifolia) guaiacol O-methyltransferase involved in the biosynthesis of veratrole, a key volatile for pollinator attraction
Source: BMC Plant Biol. 2012 Aug 31;12:158. doi: 10.1186/1471-2229-12-158 (PMC3492160; doi:10.1186/1471-2229-12-158)
Supplement: Additional file 3 — Table S2. Analysis of selection using PAML. [file 1471-2229-12-158-S3.pdf]

**Site Models**	**tree *ln* L**	ω_0	ω_1	**2δ**	***p* value**	**Significance[1]**
M0	-36389.00	0.26987	-	-	-	-
M2 branch A	-36384.09	0.26815	2.38762	9.81881	0.0017	**
M2 branch B	-36384.67	0.27258	0.14358	8.67	0.0032	**
M2 branch C	-36387.76	0.26884	1.18489	2.49165	0.114452	ns

[1]ns, not significant ($p \geq 0.05$); *, $p<0.05$; **, $p<0.01$; ***, $p<0.001$; ****, $p<0.0001$
