# Supplementary material for: Identification of white campion (Silene latifolia) guaiacol O-methyltransferase involved in the biosynthesis of veratrole, a key volatile for pollinator attraction
Source: BMC Plant Biol. 2012 Aug 31;12:158. doi: 10.1186/1471-2229-12-158 (PMC3492160; doi:10.1186/1471-2229-12-158)
Supplement: Additional file 4 — Table S3. Primers used in this study. [file 1471-2229-12-158-S4.pdf]

	Primer Sequence 5′→3′
Actin F	ACC CAA ATC ATG TTC GAG ACA TTC
Actin R	ATC ACC AGA ATC AAG CAC AAT ACC
EF1A F	CCA CAA CTC TAA CCC TAA TTT CCC
EF1A R	ATC ATA CTG ACG AGC ATC AAA CTG
CL285 F	TTC ATT GCT TGC CAC TTC TG
CL285 R	CAA ATG CGA GCT GAA AAA CA
qRT-GOMT F	CCA TGA CCC TCC AAG ACC TA
qRT-GOMT R	ACC CAT AGC TCG GTT GAA CA
Protein-SlGOMT/SdOMT-F	ATGGAAAACCCGAAAGAGCTACTAAACGC
Protein-SlGOMT/SdOMT-R	TAAGCAACGCACTTCGATGACAGACC
